# The Debate over Placebo-Controlled Trials

**DOI:** 10.1371/journal.pmed.0020157

**Published:** 2005-06-28

**Authors:** Frankllin Miller

**Affiliations:** National Institutes of HealthBethesda, MarylandUnited States of America

Turner and Tramèr provide a cogent argument in favor of the ethical use of placebo controls despite “proven effective treatment” [1]. However, they are wide of the mark citing the APPROVe trial in support of their position. Because there is no established treatment to prevent adenomatous polyps, few commentators would have any objections to the use of placebo controls in this study. Nevertheless, they are right to suggest that it would have been desirable to have included a placebo control in the VIGOR study to provide a more rigorous assessment of safety. Whether, all things considered, a placebo control would have been ethical in this study of treatment for rheumatoid arthritis is debatable.

Another issue not discussed in this *PLoS Medicine* Debate is the value of placebo controls in early “proof of concept” efficacy trials, despite the existence of established treatment. The efficiency of seeking a rigorous efficacy signal before moving on to larger-scale trials (and exposing as few subjects as possible to drugs that might not work or turn out to be toxic) is a valid ethical reason for using placebo controls, provided subjects are not exposed to undue risks of harm from withholding established treatment [2].
